# Association between the Osteoporosis Self-Assessment Tool for Asians Score and Mortality in Patients with Isolated Moderate and Severe Traumatic Brain Injury: A Propensity Score-Matched Analysis

**DOI:** 10.3390/ijerph13121203

**Published:** 2016-12-03

**Authors:** Cheng-Shyuan Rau, Pao-Jen Kuo, Shao-Chun Wu, Yi-Chun Chen, Hsiao-Yun Hsieh, Ching-Hua Hsieh

**Affiliations:** 1 Department of Neurosurgery, Kaohsiung Chang Gung Memorial Hospital, Kaohsiung 83301, Taiwan; ersh2127@cloud.cgmh.org.tw; 2 Chang Gung University College of Medicine, Taoyuan City 33302, Taiwan; bow110470@gmail.com (P.-J.K.); shaochunwu@gmail.com (S.-C.W.); libe320@yahoo.com.tw (Y.-C.C.); sylvia19870714@hotmail.com (H.-Y.H.); 3 Department of Plastic Surgery, Kaohsiung Chang Gung Memorial Hospital, Kaohsiung 83301, Taiwan; 4 Department of Anesthesiology, Kaohsiung Chang Gung Memorial Hospital, Kaohsiung 83301, Taiwan

**Keywords:** Osteoporosis Self-Assessment Tool for Asians (OSTA), mortality, traumatic brain injury (TBI), injury severity score (ISS), osteoporosis

## Abstract

*Background:* The purpose of this study was to use a propensity score-matched analysis to investigate the association between the Osteoporosis Self-Assessment Tool for Asians (OSTA) scores and clinical outcomes of patients with isolated moderate and severe traumatic brain injury (TBI). *Methods:* The study population comprised 7855 patients aged ≥40 years who were hospitalized for treatment of isolated moderate and severe TBI (an Abbreviated Injury Scale (AIS) ≥3 points only in the head and not in other regions of the body) between 1 January 2009 and 31 December 2014. Patients were categorized as high-risk (OSTA score < −4; *n* = 849), medium-risk (−4 ≤ OSTA score ≤ −1; *n* = 1647), or low-risk (OSTA score > −1; *n* = 5359). Two-sided Pearson’s chi-squared, or Fisher’s exact tests were used to compare categorical data. Unpaired Student’s *t*-test and Mann-Whitney U test were performed to analyze normally and non-normally distributed continuous data, respectively. Propensity score-matching in a 1:1 ratio was performed using NCSS software, with adjustment for covariates. *Results:* Compared to low-risk patients, high- and medium-risk patients were significantly older and injured more severely. The high- and medium-risk patients had significantly higher mortality rates, longer hospital length of stay, and a higher proportion of admission to the intensive care unit than low-risk patients. Analysis of propensity score-matched patients with adjusted covariates, including gender, co-morbidity, blood alcohol concentration level, Glasgow Coma Scale score, and Injury Severity Score revealed that high- and medium-risk patients still had a 2.4-fold (odds ratio (OR), 2.4; 95% confidence interval (CI), 1.39–4.15; *p* = 0.001) and 1.8-fold (OR, 1.8; 95% CI, 1.19–2.86; *p* = 0.005) higher mortality, respectively, than low-risk patients. However, further addition of age as a covariate for the propensity score-matching demonstrated that there was no significant difference between high-risk and low-risk patients or between medium-risk and low-risk patients, implying that older age may contribute to the significantly higher mortality associated with a lower OSTA score. *Conclusions:* Older age may be able to explain the association of lower OSTA score and higher mortality rates in patients with isolated moderate and severe TBI.

## 1. Introduction

Osteoporosis causes increased susceptibility to fragility fractures and has rapidly become a growing global health issue as the elderly population increases [1]. It is projected that the total burden of osteoporosis will grow by almost 50%, from >2 million fractures in 2005 to >3 million fractures by 2025 [2]. To identify women at risk for osteoporosis in the Asian region, the World Health Organization (WHO) developed the Osteoporosis Self-Assessment Tool for Asians (OSTA) score, which is calculated using the following formula: (body weight (kg) − age (years)) × 0.2 [3]. Three categories of patients were arbitrarily created using the index: patients at high-risk (OSTA score < −4), medium-risk (−4 ≤ OSTA score ≤ −1), or low-risk (OSTA score > −1) for osteoporosis [4,5]. The risk of osteoporosis in the high-, medium- and low- risk patients was found to be 61%, 15% and 3%, respectively [3,6]. The OSTA score has a significant positive correlation with the T-scores of bone mineral density as measured by dual-energy X-ray absorptiometry [7,8], and has been validated as an effective and feasible screening method in many Asian countries [3,6,7,9,10,11,12,13,14] to identify both men and women [4,8,15,16] with low bone mineral density who are at risk for osteoporosis.

Traumatic brain injury (TBI) accounts for about 30% of all injury deaths and is a major cause of death in the United States [17]. TBI is also a major cause of disability and death worldwide, particularly among older adults [18]. A recent study indicated that a lower OSTA score is an independent risk factor for TBI and is an indication of poorer recovery in patients with isolated, moderate TBI [19]. The osteoporosis and the recovery from TBI have multiple risk factors in common, such as age, estrogen, and vitamin D [19], however, the association between lower OSTA and the recovery of TBI is unknown. Furthermore, whether lower OSTA scores are associated with higher mortality in TBI patients is unexplored. In this study, we hypothesized that the patients with a lower OSTA score are associated with a worse outcome following a moderate or severe TBI. To avoid the confounding effects of other injuries and risk factors, this study investigated the association between OSTA score and mortality among cases of isolated moderate and severe TBI from a Level I trauma center by using propensity score-matched analysis.

## 2. Methods

### Study Design

**Figure 1:**
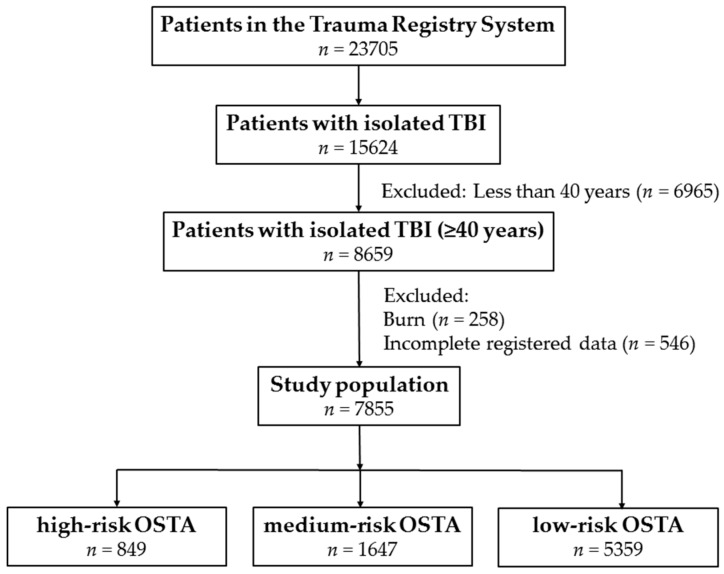
Flow chart of the studied trauma patients with isolated moderate and severe TBI. TBI: traumatic brain injury; OSTA: Osteoporosis Self-Assessment Tool for Asians.

This study was approved by the Institutional Review Board (IRB) of the Chang Gung Memorial Hospital with the approval number 201600348B0. The informed consent was waived according to the hospital IRB regulations. In this study, the patient cohort included those who were ≥40 years of age and hospitalized for treatment of moderate and severe TBI, defined as an Abbreviated Injury Scale (AIS) score ≥3 points in the head (moderate TBI, AIS 3–4; severe TBI, AIS 5) [20]. To avoid the confounding effect of injuries to other body regions, the polytrauma patients [21] who had additional AIS scores ≥3 points in any other region of the body were excluded from the study. The OSTA score was calculated based on age (years) and body weight (kilogram), using the following formula: (body weight (kg) – age (year)) × 0.2 [3]. Patients who had sustained a burn injury (*n* = 258) or whose registered data were incomplete (*n* = 546) were excluded. Among all 23,705 patients who were enrolled in the Trauma Registry System between 1 January 2009 and 31 December 2015 (Figure 1), a total of 7855 patients were included in the study population. The population consisted of (1) 849 high-risk patients (OSTA score < −4), (2) 1647 medium-risk patients (−4 ≤ OSTA score ≤ −1), and (3) 5359 low-risk patients (OSTA score > −1). The patient information retrieved from the Trauma Registry System included: age; gender; co-morbidities such as diabetes mellitus (DM), hypertension (HTN), coronary artery disease (CAD), congestive heart failure (CHF), cerebral vascular accident (CVA), and end-stage renal disease (ESRD); blood alcohol concentration (BAC) level; Glasgow Coma Scale (GCS) score upon arrival to the emergency department (ED); Injury Severity Score (ISS); TBI-related diagnoses such as epidural hematoma (EDH), subdural hematoma (SDH), subarachnoid hemorrhage (SAH), intracerebral hematoma (ICH), and cerebral contusion; in-hospital mortality; length of stay (LOS) in hospital; and the number of patients admitted into the intensive care unit (ICU). For the patients who died during the hospitalization, their lengths of stay were included in the analysis. The ISS is expressed as the median and interquartile range (IQR) with first to third quartile (Q1–Q3). The IBM SPSS Statistics for Windows, v. 20.0 (IBM Corp., Armonk, NY, USA) was used for data collection and analysis. Categorical data were compared by two-sided Pearson’s chi-squared test or Fisher’s exact test. Odds ratios (ORs) of the associated conditions and injuries of the patients were calculated with 95% confidence intervals (CIs). The continuous data were expressed as mean ± standard deviation. Continuous data with normal and non-normal distribution were analyzed with unpaired Student’s *t*-test and Mann-Whitney U test, respectively. The primary outcome was in-hospital mortality. The secondary outcomes were LOS in hospital and proportion of patients requiring admission to the ICU. To minimize the confounding effects of non-randomized assignment of OSTA grouping on the outcome assessment, NCSS software v. 10 (NCSS Statistical Software, Kaysville, UT, USA) was used to calculate the propensity score with adjustments for patient age, gender, co-morbidities, BAC, GCS, and ISS. According to the calculated propensity score, a separate 1:1 matched set of comparable study populations for the high-risk vs. low-risk and the medium-risk vs. low-risk patients was created by the greedy method and a 0.2 caliper width. Conditional logistic regression was performed to assess the effect of OSTA-related grouping on the patient outcomes. *p*-values < 0.05 were considered statistically significant.

## 3. Results

### 3.1. Characteristics and Injury Severity of Patients

The mean age of high- and medium-risk patients was significantly higher than that of low-risk patients (Table 1). There were significantly more female patients in the high- and medium-risk patient groups than in the low-risk patient group. Both high- and medium-risk patients had higher odds of DM, HTN, CAD, and CVA than low-risk patients. High-risk patients, but not medium-risk patients, also had higher odds of CHF and ESRD than low-risk patients. In addition, a significantly lower level of BAC and incidence of BAC ≥50 mg/dL was found in both high- and medium-risk patients compared to low-risk patients. GCS scores were significantly lower both in high- and medium-risk patients compared to those of low-risk patients, with a significantly higher incidence of GCS ≤8 and between 9–12, but a lower incidence of GCS ≥13 in high- and medium-risk patients. A significantly lower ISS was found in high-risk patients and medium-risk patients than in low-risk patients. When stratified by ISS (<16, 16–24 or ≥25), fewer high-risk and medium-risk patients had an ISS of <16 compared to low-risk patients. In contrast, more high-risk patients and medium-risk patients had an ISS of 16–24 or an ISS ≥25 than low-risk patients.

### 3.2. Patient Outcomes

Compared to low-risk patients, high- and medium-risk patients had a 5.3-fold (OR, 5.3; 95% CI, 3.76–7.49; *p* < 0.001) and 3.1-fold (OR, 3.1; 95% CI, 2.23–4.29; *p* < 0.001) higher mortality, respectively. A significantly longer LOS in hospital was found in high-risk patients (mean, 9.8 days) and medium-risk patients (mean, 9.0 days) than in low-risk patients (mean, 8.2 days). In addition, a significantly higher proportion of high-risk (38.5%) and medium-risk patients were admitted to the ICU (23.9%) relative to low-risk patients (14.9%). There was no significant difference in rates of EDH between high-risk and low-risk patients or between medium-risk and low-risk patients. However, high-risk and medium-risk patients had greater odds of SDH (3.7 and 1.9, respectively), SAH (1.6 and 1.3, respectively), ICH (3.0 and 1.4, respectively), and cerebral contusion (2.7 and 1.6, respectively) than low-risk patients.

Regarding the fatality of different types of trauma injury to the brain including EDH, SDH, SAH, ICH, and cerebral contusion (Table 2), both high-risk patients and medium-risk patients had higher odds of mortality than low-risk patients. However, the higher odds were only significant in high-risk patients who sustained EDH, SDH, or cerebral contusion and in medium-risk patients who sustained EDH, SDH, SAH, or ICH.

### 3.3. Outcome of Propensity Score-Matched Analysis

A separate set of propensity score-matched comparable study populations for high- and medium-risk vs. low-risk patients, respectively, was created for the comparison of OSTA score effect on the outcome. In these pairs of propensity score-matched patients, there was no significant difference in gender, co-morbidity, BAC, GCS, and ISS. Because age is an inherent component in the calculation of OSTA score, it was not included as an adjusted covariate at the beginning of propensity score-matched analysis. The primary and secondary outcome was compared in the 756 well-balanced pairs of high-risk and low-risk patients (Table 3) and 1570 well-balanced pairs of medium-risk and low-risk patients (Table 4).

The high- and medium-risk patients still had a 2.4-fold (OR, 2.4; 95% CI, 1.39–4.15; *p* = 0.001) and 1.8-fold (OR, 1.8; 95% CI, 1.19–2.86; *p* = 0.005) higher mortality, respectively, than low-risk patients. However, the high- and medium-risk patients did not differ significantly in terms of hospital LOS or the proportion of ICU admission, implying that the aforementioned difference in hospital LOS and proportion of ICU admission observed between high-risk and low-risk patients as well as between medium-risk and low risk patients may be attributed to gender, pre-existing co-morbidities, and associated injury severity. Regarding the fatality of different types of trauma injury to the brain, which included EDH, SDH, SAH, ICH, and cerebral contusion, although both high-risk patients (Table 5) and medium-risk patients (Table 6) in the propensity score-matched patient population had a higher odds of mortality than low-risk patients, none of the differences were statistically significant. This implies that the higher odds of mortality in high- and medium-risk patients compared to that in low-risk patients could not be explained only by the trauma injury to the brain. However, further addition of age as an adjusted covariate for the propensity score-matched groups of patients demonstrated that there were no significant differences of ICU admission between high-risk and low-risk patients (OR, 1.4; 95% CI, 0.89–2.07; *p* = 0.160) (Table 7) or between medium-risk and low-risk patients (OR, 1.2; 95% CI, 0.95–1.47; *p* = 0.145) (Table 8). These findings imply that older age may contribute to the significantly higher mortality associated with a lower OSTA score.

## 4. Discussion

This study compared the clinical outcome of patients hospitalized for isolated moderate and severe TBI with the risk of osteoporosis as determined by OSTA score. Compared to low-risk patients, high- and medium-risk patients were significantly older and predominantly female; presented with higher incidences of co-morbidity; were injured more severely; and had greater odds of SDH, SAH, ICH, and cerebral contusion. The high- and medium-risk patients had significantly higher mortality rate, longer hospital LOS, and higher proportion of patients admitted to the ICU than low-risk patients. To reduce bias caused by confounding from non-random assignment of baseline covariates, including gender, co-morbidity, BAC, GCS, and ISS, well-balanced propensity score-matched patients were selected for analysis. The analysis revealed that high- and medium-risk patients still had a higher mortality than low-risk patients. However, further addition of age as an adjusted covariate for the propensity score-matching demonstrated that there were no significant differences between high-risk and low-risk patients or between medium-risk and low-risk patients, implying older age may contribute to the significantly higher mortality observed among high- and medium-risk patients.

To reduce the bias caused by confounding factors in observational studies, the propensity score analysis has become a common method for adjusting for confounders [22]. As a balancing score, the propensity score method helps to assign a conditional distribution of pre-exposure characteristics, given that the propensity score is the same for the exposed and unexposed groups [23]. In this study, age, gender, co-morbidity, BAC, GCS, and ISS were used as covariates for the propensity score-matching. In addition to being comorbidities, GCS and ISS have been well recognized as independent risk factors for mortality due to TBI [24,25,26,27]; gender and BAC also may have a significant effect on the mortality of patients who sustained TBI [28,29]. In a retrospective study of 1627 TBI patients, female patients had a significantly higher mortality (3.4% vs. 1.6%, *p* = 0.048) [30]. Patients who consumed alcohol tended to experience lower moderate-to-severe TBI injuries [31]. Furthermore, some studies demonstrated a beneficial effect of alcohol on patients with TBI, which could not be explained by the associated lower injury severity to the brain [32,33]. In this study, higher-risk and medium-risk patients, with adjusted estimate of covariates, had 2.4-fold and 1.8-fold higher mortality, respectively, than low-risk patients. However, no significant difference in mortality was found regarding the mortality rates associated with different types of trauma injury to the brain, implying that the higher odds of mortality in high- and medium-risk patients compared to that in low-risk patients could not be explained only by the effect of trauma injury to the brain.

Furthermore, in addition to TBI, age is a strong, independent prognostic factor [34,35]. Mortality rates of patients who sustained TBI with AIS ≥ 3 is higher in the geriatric population independent of the type of head trauma injury [34]. Following TBI, varied ages had been reported as the critical threshold from 35 [36], 40 [37], 50 [38], or 60 [39] years for an associated poor prognosis. Even in terms of late mortality, in a multivariate Cox regression analysis of 2545 patients who received rehabilitation for severe TBI and were discharged alive, an age ≥35 years was the strongest predictor of mortality, with a nearly threefold increased risk of mortality reported than those who had an age less than 35 years [40]. In this study, it is expected that high- and medium-risk patients were older and thus had a higher mortality than low-risk patients. When the comparison was made between the propensity score-matched patients, adjusted for age and other covariates, no significant difference was found between high-risk and low-risk patients or between medium-risk and low-risk patients, implying older age contributed to the association of lower OSTA score with higher mortality among patients with isolated moderate and severe TBI.

In this study, we did not find that a lower OSTA score would contribute to a higher mortality in patients with isolated moderate and severe TBI. This result seemed to be contradictory to the results reported by Chao et al., which stated that a lower OSTA score acts as an independent risk factor and predicts poorer recovery in patients with isolated, moderate TBI [19]. However, the study used the Glasgow outcome score (GOS) as the primary outcome and they compared groups who had a good recovery (GOS of 5) and inadequate recovery (GOS 1–4) with moderate TBI patients who had GCS scores between 9 and 13 [19]. In contrast, our study evaluated the association between isolated TBI with AIS scores ≥3 points in the head with mortality (a more detrimental result) as the outcome measurement. In addition, their study only included 107 patients with isolated, moderate TBI and used univariate and multivariate logistic regression for the analysis. In their study, younger age was recognized to be associated with better outcomes in the univariate, but not multivariate logistic regression. In contrast, another study found that a higher OSTA score was a risk factor for predicting a good recovery using univariate and multivariate analysis [19]. Obviously, as a major component in the calculation of OSTA score, age was intrinsically linked to the OSTA score in the statistical analysis. In our study, a total of 7855 patients were enrolled in the study group and the analysis was performed using the propensity score-matching to attenuate the baseline confounding effect. In this study, the older age may be able to explain the association of lower OSTA scores with higher mortality in patients with isolated moderate and severe TBI. Furthermore, in the age-included propensity score-matched analysis, in contrast to a higher risk of mortality (OR, 1.5; 95% CI, 0.82–2.60) for medium-risk patients than low-risk patients, a lower risk of mortality (OR, 0.7; 95% CI, 0.23–1.88) for high-risk patients than low-risk patients was even found, albeit there were statistically non-significant. Notably, in the 756 pairs of age-not-included propensity score-matched analysis, the high-risk patients had a 2.4-fold (OR, 2.4; 95% CI, 1.39–4.15) higher mortality than low-risk patients, but in the age-included propensity score-matching only 203 pairs of high-risk vs. low-risk patients were analyzed, the number of pairs of high-risk vs. low-risk patients (*n* = 203) in the age-included propensity score-matched analysis is far less than that of the pairs of patients (*n* = 756) in the age-not-included propensity score-matching and may present as a selection bias. We suspected the above lower risk of mortality of high-risk vs. low-risk patients may be attributed to a selection bias, seeing the low-risk patients selected for matched analysis would be much older and comprise only some portion of the low-risk study population.

Our study has some other limitations that should be acknowledged. First, there is an inherent selection bias because of the retrospective design. Second, the descriptive study lacked important information regarding the indication and type of surgery performed on patients, thus we could only assume that there was uniform assessment and management of patients. Third, discrimination of age and other covariates are obvious between high-risk, medium-risk, and low-risk patients. However, excellent discrimination of the propensity score model could lead to little or even no overlap of the estimated propensity score between the exposure and control groups. Propensity score-matched 203 and 1055 pairs of patients were created, respectively, adjusting for age and other covariates. When using propensity scores with high discrimination to create a matched sample, there would be a small number of exposure and control group subjects that could be matched, leaving few subjects in the study sample for further comparison [22]. Using a propensity score estimated from a model with very good or excellent discrimination, the estimates of treatment effect could be biased. Fourth, the patients declared dead on hospital arrival or at the accident scene were not included in the Trauma Registry Database, which may have led to bias. Finally, aside from mortality, it is important to evaluate other outcomes, such as medical, socioeconomic, and rehabilitation measures, which could not be included in this study.

## 5. Conclusions

The propensity score-matched analysis revealed that older age may be able to explain the association of lower OSTA scores with higher mortality in patients with isolated moderate and severe TBI.

## Figures and Tables

**Table 1:** Demographics and injury characteristics of high-risk, medium-risk, and low-risk patients with isolated moderate and severe traumatic brain injury (TBI).

Variables	High Risk OSTA < −4 *n* = 849 (I)	Medium Risk −4 ≤ OSTA ≤ −1 *n* = 1647 (II)	Low Risk OSTA > −1 *n* = 5359 (III)	Odds Ratio (95% CI) I vs. III	*p*	Odds Ratio (95% CI) II vs. III	*p*
**Age**	79.7 ± 6.9	68.1 ± 8.0	54.1 ± 8.8	-	<0.001	-	<0.001
**Gender**					<0.001		<0.001
**Female**	550 (64.8)	1015 (61.6)	2071 (38.6)	2.9 (2.51–3.40)		2.6 (2.28–2.86)	
**Male**	299 (35.2)	632 (38.4)	3288 (61.4)	0.3 (0.29–0.40)		0.4 (0.35–0.44)	
**Co-Morbidity**							
**Diabetes mellitus (DM)**	155 (18.3)	397 (24.1)	800 (14.9)	1.3 (1.05–1.54)	0.013	1.8 (1.58–2.07)	<0.001
**Hypertension (HTN)**	440 (51.8)	720 (43.7)	1490 (27.8)	2.8 (2.41–3.24)	<0.001	2.0 (1.80–2.26)	<0.001
**Coronary artery disease (CAD)**	70 (8.2)	96 (5.8)	146 (2.7)	3.2 (2.39–4.31)	<0.001	2.2 (1.70–2.88)	<0.001
**Congestive heart failure (CHF)**	22 (2.6)	18 (1.1)	36 (0.7)	3.9 (2.30–6.72)	<0.001	1.6 (0.93–2.89)	0.087
**Cerebral vascular accident (CVA)**	87 (10.2)	128 (7.8)	112 (2.1)	5.3 (4.00–7.15)	<0.001	3.9 (3.04–5.12)	<0.001
**End-stage renal disease (ESRD)**	4 (0.5)	4 (0.2)	3 (0.1)	8.5 (1.89–37.83)	0.009	4.3 (0.97–19.44)	0.058
**Blood alcohol concentration (BAC) (mg/dL)**	0.9 ± 13.0	2.9 ± 23.5	14.5 ± 55.7	-	<0.001	-	<0.001
**BAC ≥ 50 mg/dL, *n* (%)**	5 (0.6)	27 (1.6)	375 (7.0)	0.1 (0.03–0.19)	<0.001	0.2 (0.15–0.33)	<0.001
**Glasgow coma scale (GCS)**	13.7 ± 2.8	14.1 ± 2.5	14.4 ± 2.1	-	<0.001	-	<0.001
**≤8**	70 (8.2)	93 (5.6)	230 (4.3)	2.0 (1.52–2.65)	<0.001	1.3 (1.04–1.71)	0.022
**9–12**	63 (7.4)	85 (5.2)	156 (2.9)	2.7 (1.98–3.62)	<0.001	1.8 (1.39–2.38)	<0.001
**≥13**	716 (84.3)	1469 (89.2)	4973 (92.8)	0.4 (0.34–0.52)	<0.001	0.6 (0.53–0.77)	<0.001
**Injury severity score (ISS), median (interquartile range (IQR))**	8 (4–16)	4 (4–12)	4 (4–8)	-	<0.001	-	<0.001
**<16**	553 (65.1)	1287 (78.1)	4682 (87.4)	0.3 (0.23–0.32)	<0.001	0.5 (0.45–0.60)	<0.001
**16–24**	238 (28.0)	288 (17.5)	555 (10.4)	3.4 (2.83–4.01)	<0.001	1.8 (1.57–2.14)	<0.001
**≥25**	58 (6.8)	72 (4.4)	122 (2.3)	3.1 (2.28–4.34)	<0.001	2.0 (1.46–2.64)	<0.001
**Mortality, *n* (%)**	61 (7.2)	71 (4.3)	77 (1.4)	5.3 (3.76–7.49)	<0.001	3.1 (2.23–4.29)	<0.001
**Length of stay (LOS) in hospital (days)**	9.8 ± 11.2	9.0 ± 10.5	8.2 ± 9.0	-	<0.001	-	0.010
**Intensive care unit (ICU) admission, *n* (%)**	325 (38.3)	393 (23.9)	796 (14.9)	3.6 (3.04–4.16)	<0.001	1.8 (1.57–2.06)	<0.001
**Head trauma, *n* (%)**							
**Epidural hematoma (EDH)**	45 (5.3)	53 (3.2)	219 (4.1)	1.3 (0.95–1.83)	0.103	0.8 (0.58–1.06)	0.110
**Subdural hematoma (SDH)**	259 (30.5)	309 (18.8)	570 (10.6)	3.7 (3.11–4.37)	<0.001	1.9 (1.67–2.26)	<0.001
**Subarachnoid hemorrhage (SAH)**	137 (16.1)	231 (14.0)	588 (11.0)	1.6 (1.28–1.91)	<0.001	1.3 (1.12–1.56)	0.001
**Intracerebral hematoma (ICH)**	59 (6.9)	57 (3.5)	130 (2.4)	3.0 (2.19–4.12)	<0.001	1.4 (1.05–1.98)	0.023
**Cerebral contusion**	116 (13.7)	144 (8.7)	296 (5.5)	2.7 (2.16–3.40)	<0.001	1.6 (1.33–2.02)	<0.001

**Table 2:** Mortality rate of different types of trauma injury to the brain in high-risk, medium-risk, and low-risk patients.

Mortality Rate	High Risk OSTA < −4 *n* = 849 (I)	Medium Risk −4 ≤ OSTA ≤ −1 *n* = 1647 (II)	Low Risk OSTA > −1 *n* = 5359 (III)	Odds Ratio (95% CI) I vs. III	*p*	Odds Ratio (95% CI) II vs. III	*p*
**Head trauma, *n* (%)**							
**Epidural hematoma (EDH)**	10/45 (22.2)	12/53 (22.6)	22/219 (10.0)	2.6 (1.12–5.86)	0.023	2.6 (1.20–5.72)	0.013
**Subdural hematoma (SDH)**	39/259 (15.1)	50/309 (16.2)	52/570 (9.1)	1.8 (1.13–2.75)	0.011	1.9 (1.27–2.92)	0.002
**Subarachnoid hemorrhage (SAH)**	13/137 (9.5)	31/231 (13.4)	31/588 (5.3)	1.9 (0.96–3.70)	0.063	2.8 (1.65–4.70)	<0.001
**Intracerebral hematoma (ICH)**	10/59 (16.9)	11/57 (19.3)	11/130 (8.5)	2.2 (0.88–5.53)	0.085	2.6 (1.05–6.38)	0.034
**Cerebral contusion**	22/116 (19.0)	12/144 (8.3)	14/296 (4.7)	4.7 (2.32–9.59)	<0.001	1.8 (0.82–4.07)	0.133

**Table 3:** Outcome of the high-risk and low-risk patients before and after propensity score-matching with adjustment of the covariates including gender, co-morbidity, BAC, GCS, and ISS.

Variables	Before Matching				After Matching			
	High Risk OSTA < −4 *n* = 849	Low Risk OSTA > −1 *n* = 5359	Odds Ratio (95% CI)	*p*	High Risk OSTA < −4 *n* = 756	Low Risk OSTA > −1 *n* = 756	Odds Ratio (95% CI)	*p*
**Gender**								
**Female**	550 (64.8)	2071 (38.6)	2.9 (2.51–3.40)	<0.001	474 (62.7)	474 (62.7)	1.0 (0.81–1.23)	1.000
**Male**	299 (35.2)	3288 (61.4)	0.3 (0.29–0.40)		282 (37.3)	282 (37.3)	1.0 (0.81–1.23)	
**Co-Morbidity**								
**DM**	155 (18.3)	800 (14.9)	1.3 (1.05–1.54)	0.013	131 (17.3)	131 (17.3)	1.0 (0.77–1.31)	1.000
**HTN**	440 (51.8)	1490 (27.8)	2.8 (2.41–3.24)	<0.001	372 (49.2)	372 (49.2)	1.0 (0.82–1.22)	1.000
**CAD**	70 (8.2)	146 (2.7)	3.2 (2.39–4.31)	<0.001	41 (5.4)	41 (5.4)	1.0 (0.64–1.56)	1.000
**CHF**	22 (2.6)	36 (0.7)	3.9 (2.30–6.72)	<0.001	8 (1.1)	8 (1.1)	1.0 (0.37–2.68)	1.000
**CVA**	87 (10.2)	112 (2.1)	5.3 (4.00–7.15)	<0.001	49 (6.5)	49 (6.5)	1.0 (0.66–1.51)	1.000
**ESRD**	4 (0.5)	3 (0.1)	8.5 (1.89–37.83)	0.009	0 (0.0)	0 (0.0)	-	-
**BAC (mg/dL)**	0.9 ± 13.0	14.5 ± 55.7	-	<0.001	0.8 ± 11.9	0.7 ± 10.7	-	0.881
**GCS**	13.7 ± 2.8	14.4 ± 2.1	-	<0.001	14.0 ± 2.5	14.0 ± 2.5	-	0.560
**ISS, median (IQR)**	8 (4–16)	4 (4–8)	-	<0.001	6 (4–16)	6 (4–16)	-	0.889
**Mortality, *n* (%)**	61 (7.2)	77 (1.4)	5.3 (3.76–7.49)	<0.001	44 (5.8)	19 (2.5)	2.4 (1.39–4.15)	0.001
**LOS in hospital (days)**	9.8 ± 11.2	8.2 ± 9.0	-	<0.001	9.4 ± 10.7	10.2 ± 10.5	-	0.131
**ICU admission, *n* (%)**	325 (38.3)	796 (14.9)	3.6 (3.04–4.16)	<0.001	264 (34.9)	256 (33.9)	1.0 (0.85–1.30)	0.665

**Table 4:** Outcome of medium-risk and low-risk patients before and after propensity score-matching with adjustment of the covariates including gender, co-morbidity, BAC, GCS, and ISS.

Variables	Before Matching				After Matching			
	Medium Risk −4 ≤ OSTA ≤ −1 *n* = 1647	Low Risk OSTA > −1 *n* = 5359	Odds Ratio (95% CI)	*p*	Medium Risk −4 ≤ OSTA ≤ −1 *n* = 1570	Low Risk OSTA > −1 *n* = 1570	Odds Ratio (95% CI)	*p*
**Gender**								
**Female**	1015 (61.6)	2071 (38.6)	2.6 (2.28–2.86)	<0.001	974 (62.0)	974 (62.0)	1.0 (0.87–1.16)	1.000
**Male**	632 (38.4)	3288 (61.4)	0.4 (0.35–0.44)		596 (38.0)	596 (38.0)	1.0 (0.87–1.16)	
**Co-Morbidity**								
**DM**	397 (24.1)	800 (14.9)	1.8 (1.58–2.07)	<0.001	364 (23.2)	364 (23.2)	1.0 (0.85–1.18)	1.000
**HTN**	720 (43.7)	1490 (27.8)	2.0 (1.80–2.26)	<0.001	68 (42.5)	68 (42.5)	1.0 (0.87–1.15)	1.000
**CAD**	96 (5.8)	146 (2.7)	2.2 (1.70–2.88)	<0.001	64 (4.1)	64 (4.1)	1.0 (0.70–1.42)	1.000
**CHF**	18 (1.1)	36 (0.7)	1.6 (0.93–2.89)	0.087	6 (0.4)	6 (0.4)	1.0 (0.32–3.11)	1.000
**CVA**	128 (7.8)	112 (2.1)	3.9 (3.04–5.12)	<0.001	83 (5.3)	83 (5.3)	1.0 (0.73–1.37)	1.000
**ESRD**	4 (0.2)	3 (0.1)	4.3 (0.97–19.44)	0.058	0 (0.0)	0 (0.0)	-	-
**BAC (mg/dL)**	2.9 ± 23.5	14.5 ± 55.7	-	<0.001	2.9 ± 23.5	2.9 ± 23.8	-	0.956
**GCS**	14.1 ± 2.5	14.4 ± 2.1	-	<0.001	14.2 ± 2.4	14.3 ± 2.2	-	0.336
**ISS, median (IQR)**	4 (4–12)	4 (4–8)	-	<0.001	4 (4–10)	4 (4–10)	-	0.754
**Mortality, *n* (%)**	71 (4.3)	77 (1.4)	3.1 (2.23–4.29)	<0.001	58 (3.7)	32 (2.0)	1.8 (1.19–2.86)	0.005
**LOS in hospital (days)**	9.0 ± 10.5	8.2 ± 9.0	-	0.010	8.9 ± 10.1	9.2 ± 10.0	-	0.429
**ICU admission, *n* (%)**	393 (23.9)	796 (14.9)	1.8 (1.57–2.06)	<0.001	360 (22.9)	348 (22.2)	1.0 (0.88–1.24)	0.608
**LOS in ICU (days)**	8.8 ± 12.5	7.2 ± 8.9	-	0.027	8.5 ± 12.2	7.3 ± 8.1	-	0.123

**Table 5:** Mortality rate of different types of trauma injury to the brain in high-risk vs. low-risk patients.

Mortality Rate	Before Matching				After Matching			
	High Risk OSTA < −4 *n* = 849	Low Risk OSTA > −1 *n* = 5359	Odds Ratio (95% CI)	*p*	High Risk OSTA < −4 *n* = 756	Low Risk OSTA > −1 *n* = 756	Odds Ratio (95% CI)	*p*
**Head trauma, *n* (%)**								
**Epidural hematoma (EDH)**	10/45 (22.2)	22/219 (10.0)	2.6 (1.12–5.86)	0.023	4/31 (12.9)	6/53 (11.3)	1.2 (0.30–4.48)	1.000
**Subdural hematoma (SDH)**	39/259 (15.1)	52/570 (9.1)	1.8 (1.13–2.75)	0.011	4/28 (14.3)	6/56 (10.7)	1.4 (0.36–5.39)	0.725
**Subarachnoid hemorrhage (SAH)**	13/137 (9.5)	31/588 (5.3)	1.9 (0.96–3.70)	0.063	4/25 (16.0)	7/59 (11.9)	1.4 (0.38–5.34)	0.725
**Intracerebral hematoma (ICH)**	10/59 (16.9)	11/130 (8.5)	2.2 (0.88–5.53)	0.085	7/49 (14.3)	4/35 (11.4)	1.3 (0.35–4.80)	0.756
**Cerebral contusion**	22/116 (19.0)	14/296 (4.7)	4.7 (2.32–9.59)	<0.001	3/10 (30.0)	3/15 (20.0)	1.7 (0.27–11.92)	0.653

**Table 6:** Mortality rate of different types of trauma injury to the brain in medium-risk vs. low-risk patients.

Mortality Rate	Before Matching				After Matching			
	Medium Risk −4 ≤ OSTA ≤ −1 *n* = 1647	Low Risk OSTA > −1 *n* = 5359	Odds Ratio (95% CI)	*p*	Medium Risk −4 ≤ OSTA ≤ −1 *n* = 1570	Low Risk OSTA > −1 *n* = 1570	Odds Ratio (95% CI)	*p*
**Head trauma, *n* (%)**								
**Epidural hematoma (EDH)**	12/53 (22.6)	22/219 (10.0)	2.6 (1.20–5.72)	0.013	12/50 (24.0)	10/79 (12.7)	2.2 (0.86–5.51)	0.095
**Subdural hematoma (SDH)**	50/309 (16.2)	52/570 (9.1)	1.9 (1.27–2.92)	0.002	41/280 (14.6)	24/249 (9.6)	1.6 (0.94–2.75)	0.080
**Subarachnoid hemorrhage (SAH)**	31/231 (13.4)	31/588 (5.3)	2.8 (1.65–4.70)	<0.001	27/216 (12.5)	14/223 (6.3)	2.1 (1.00–4.19)	0.052
**Intracerebral hematoma (ICH)**	11/57 (19.3)	11/130 (8.5)	2.6 (1.05–6.38)	0.034	10/53 (18.9)	6/60 (10.0)	2.1 (0.71–6.22)	0.177
**Cerebral contusion**	12/144 (8.3)	14/296 (4.7)	1.8 (0.82–4.07)	0.133	10/138 (7.2)	9/143 (6.3)	1.2 (0.46–2.96)	0.751

**Table 7:** Outcome of the high-risk and low-risk patients before and after propensity score-matching with adjustment of the covariates including age and other variables.

Variables	Before Matching				After Matching			
	High Risk OSTA < −4 *n* = 849	Low Risk OSTA > −1 *n* = 5359	Odds Ratio (95% CI)	*p*	High Risk OSTA < −4 *n* = 203	Low Risk OSTA > −1 *n* = 203	Odds Ratio (95% CI)	*p*
**Age**	79.7 ± 6.9	54.1 ± 8.8	-	<0.001	72.5 ± 5.6	72.2 ± 5.5	-	0.663
**Gender**				<0.001				1.000
**Female**	550 (64.8)	2071 (38.6)	2.9 (2.51–3.40)		120 (59.1)	120 (59.1)	1.0 (0.67–1.49)	
**Male**	299 (35.2)	3288 (61.4)	0.3 (0.29–0.40)		83 (40.9)	83 (40.9)	1.0 (0.67–1.49)	
**Co-Morbidity**								
**DM**	155 (18.3)	800 (14.9)	1.3 (1.05–1.54)	0.013	46 (22.7)	46 (22.7)	1.0 (0.63–1.59)	1.000
**HTN**	440 (51.8)	1490 (27.8)	2.8 (2.41–3.24)	<0.001	93 (45.8)	93 (45.8)	1.0 (0.68–1.48)	1.000
**CAD**	70 (8.2)	146 (2.7)	3.2 (2.39–4.31)	<0.001	14 (6.9)	14 (6.9)	1.0 (0.46–2.16)	1.000
**CHF**	22 (2.6)	36 (0.7)	3.9 (2.30–6.72)	<0.001	1 (0.5)	1 (0.5)	1.0 (0.06–16.10)	1.000
**CVA**	87 (10.2)	112 (2.1)	5.3 (4.00–7.15)	<0.001	6 (3.0)	6 (3.0)	1.0 (0.32–3.15)	1.000
**ESRD**	4 (0.5)	3 (0.1)	8.5 (1.89–37.83)	0.009	0 (0.0)	0 (0.0)	-	-
**BAC (mg/dL)**	0.9 ± 13.0	14.5 ± 55.7	-	<0.001	1.5 ± 15.0	0.0 ± 0.0	-	0.147
**GCS**	13.7 ± 2.8	14.4 ± 2.1	-	<0.001	14.2 ± 2.1	14.1 ± 2.7	-	0.625
**ISS, median (IQR)**	8 (4–16)	4 (4–8)	-	<0.001	5 (4–16)	5 (4–16)	-	0.660
**Mortality, *n* (%)**	61 (7.2)	77 (1.4)	5.3 (3.76–7.49)	<0.001	6 (3.0)	9 (4.4)	0.7 (0.23–1.88)	0.430
**LOS in hospital (days)**	9.8 ± 11.2	8.2 ± 9.0	-	<0.001	9.7 ± 9.8	10.3 ± 12.5	-	0.631
**ICU admission, *n* (%)**	325 (38.3)	796 (14.9)	3.6 (3.04–4.16)	<0.001	68 (33.5)	55 (27.1)	1.4 (0.89–2.07)	0.160

**Table 8:** Outcome of the medium-risk and low-risk patients before and after propensity score-matching with adjustment of the covariates including age and other variables.

Variables	Before Matching				After Matching			
	Medium Risk −4 ≤ OSTA ≤ −1 *n* = 1647	Low Risk OSTA > −1 *n* = 5359	Odds Ratio (95% CI)	*p*	Medium Risk −4 ≤ OSTA ≤ −1 *n* = 1055	Low Risk OSTA > −1 *n* = 1055	Odds Ratio (95% CI)	*p*
**Age**	68.1 ± 8.0	54.1 ± 8.8	-	<0.001	64.5 ± 6.8	64.4 ± 6.7	-	0.747
**Gender**				<0.001				1.000
**Female**	1015 (61.6)	2071 (38.6)	2.6 (2.28–2.86)		665 (63.0)	665 (63.0)	1.0 (0.84–1.19)	
**Male**	632 (38.4)	3288 (61.4)	0.4 (0.35–0.44)		390 (37.0)	390 (37.0)	1.0 (0.84–1.19)	
**Co-Morbidity**								
**DM**	397 (24.1)	800 (14.9)	1.8 (1.58–2.07)	<0.001	216 (20.5)	216 (20.5)	1.0 (0.81–1.24)	1.000
**HTN**	720 (43.7)	1490 (27.8)	2.0 (1.80–2.26)	<0.001	398 (37.7)	398 (37.7)	1.0 (0.84–1.19)	1.000
**CAD**	96 (5.8)	146 (2.7)	2.2 (1.70–2.88)	<0.001	37 (3.5)	37 (3.5)	1.0 (0.63–1.59)	1.000
**CHF**	18 (1.1)	36 (0.7)	1.6 (0.93–2.89)	0.087	1 (0.1)	1 (0.1)	1.0 (0.06–16.01)	1.000
**CVA**	128 (7.8)	112 (2.1)	3.9 (3.04–5.12)	<0.001	31 (2.9)	31 (2.9)	1.0 (0.60–1.66)	1.000
**ESRD**	4 (0.2)	3 (0.1)	4.3 (0.97–19.44)	0.058	0 (0.0)	0 (0.0)	-	-
**BAC (mg/dL)**	2.9 ± 23.5	14.5 ± 55.7	-	<0.001	3.7 ± 27.0	3.0 ± 25.0	-	0.506
**GCS**	14.1 ± 2.5	14.4 ± 2.1	-	<0.001	14.4 ± 2.1	14.4 ± 2.1	-	0.581
**ISS, median (IQR)**	4 (4–12)	4 (4–8)	-	<0.001	4 (4–9)	4 (4–9)	-	0.431
**Mortality, *n* (%)**	71 (4.3)	77 (1.4)	3.1 (2.23–4.29)	<0.001	29 (2.7)	20 (1.9)	1.5 (0.82–2.60)	0.193
**LOS in hospital (days)**	9.0 ± 10.5	8.2 ± 9.0	-	0.010	8.3 ± 8.9	8.5 ± 9.0	-	0.583
**ICU admission, *n* (%)**	393 (23.9)	796 (14.9)	1.8 (1.57–2.06)	<0.001	208 (19.7)	182 (17.3)	1.2 (0.95–1.47)	0.145
